# CHIP/STUB1 suppresses the transcription and latent reactivation of HIV-1 via the TRAF6-NF-κB-HIV-LTR axis

**DOI:** 10.1371/journal.ppat.1013683

**Published:** 2025-11-07

**Authors:** Wei-Hua Zheng, Sen-Ying Lin, Ze-Lan He, Run-Ze Ni, Dan Mu

**Affiliations:** 1 State Key Laboratory of Primate Biomedical Research, Kunming University of Science and Technology, Kunming, Yunnan, China; 2 Institute of Primate Translational Medicine, Kunming University of Science and Technology, Kunming, Yunnan, China; 3 Yunnan Key Laboratory of Primate Biomedical Research, Kunming, Yunnan, China; University of Illinois at Chicago College of Medicine, UNITED STATES OF AMERICA

## Abstract

HIV-1 replication, transcription and latency are correlated with the activation of NF-κB signaling. C-terminus of Hsc70-interacting protein (CHIP or STUB1), a cellular E3 ligase, has been reported to inhibit Tat-mediated HIV-1 LTR promoter activity by degrading Tat. In this study, we demonstrated that CHIP modulates HIV infection by limiting viral transcription through an uncharacterized mechanism involving the negative regulation of TRAF6-NF-κB signaling. Mechanistically, CHIP targets the NF-κB signaling transducer TRAF6 but not TRAF2 or p65 for degradation via the ubiquitin–proteasome pathway, leading to the inhibition of TRAF6-mediated NF-κB signaling, which in turn suppresses NF-κB-dependent HIV-1 LTR transcription. Notably, in addition to the U-box domain, which is well known for protein degradation, the TPR domain of CHIP plays an independent role in facilitating the proteasome-mediated degradation of TRAF6 via K48-linked polyubiquitination. Furthermore, CHIP plays an inhibitory role in the reactivation of HIV-1 latency in various models, in concert with its repressive effect on the NF-κB pathway. Thus, these findings reveal that CHIP is a novel repressor of NF-κB-driven HIV-1 promoter transactivation, providing new insights into the molecular mechanisms by which upstream NF-κB signaling influences HIV-1 replication, transcription and latency.

## Author summary

The constant latency of human immunodeficiency virus type 1 (HIV-1) is the main barrier to eradicating the virus from infected individuals by current therapeutics. Host factors involved in HIV LTR promoter transcription regulation through NF-κB signaling are far from fully understood. In this manuscript, we demonstrated that the C-terminus of Hsc70-interacting protein (CHIP or STUB1) suppresses HIV-1 postintegration transcription and facilitates the maintenance of viral latency via the TRAF6-NF-κB-HIV-LTR axis. We found that CHIP targets the NF-κB signaling transducer TRAF6 for proteasome-mediated degradation via K48-linked polyubiquitination, leading to the inhibition of TRAF6-mediated NF-κB signaling and the suppression of NF-κB-dependent HIV-1 LTR-driven transcription. We demonstrated that the TPR domain together with the U-box domain of CHIP play critical roles in facilitating the proteasome-mediated degradation of TRAF6. Overall, these results show that CHIP influences HIV expression and latency. CHIP may be a useful new target for reactivating HIV gene expression in people with HIV.

## Introduction

The constant latency of human immunodeficiency virus type 1 (HIV-1) is the main barrier to eradicating the virus from infected individuals by current therapeutics [1,2]. HIV latency results from inadequate postintegration gene expression, which is determined by the activity of the enhancer and promoter elements within the HIV-1 5’ end long terminal repeat (LTR). Factors that have been proposed to regulate the transcriptional activity of the HIV-1 LTR include 1) the inadequate availability of signal-responsive transcription factors such as NF-κB, Sp1, P-TEFb or CDK9/CycinT1, and HIV-1 Tat; 2) specific transcriptional repressors; and 3) epigenetic regulatory factors that bind to the HIV-1 LTR [3,4]. However, the mechanism of HIV postintegration transcriptional repression is far from fully understood.

NF-κB plays a critical role in efficient HIV transcription by binding to two adjacent κB sites within the LTR promoter. The activation of NF-κB involves two major signaling pathways: the canonical and noncanonical (or alternative) pathways. The canonical activation of NF-κB signaling involves IκB kinase (IKK) activation and subsequent IκBα degradation, resulting in the rapid and transient nuclear translocation of NF-κB p65, which binds and activates the cis-acting regulatory elements of target genes [5]. TNF receptor-associated factor (TRAF) 2, 5, and 6 are involved in mediating the phosphorylation and activation of transforming growth factor beta-activated kinase 1 (TAK1), which subsequently activates IKK, leading to NF-κB nuclear translocation [6].

Much is known about the downstream NF-κB signaling regulation. Our group and others have demonstrated that various proteins regulate the recruitment of NF-κB to the HIV-1 LTR, thus affecting LTR-mediated viral transcription [7–10]. Few upstream NF-κB signaling pathways have been investigated in the context of the regulation of HIV transcription. Among them, CYLD controls HIV transcription by inhibiting NF-κB nuclear translocation [9]; PEBP1 suppresses HIV transcription and induces latency by inactivating MAPK/NF-κB signaling [11]; and the ubiquitin ligase BIRC2/cIAP1, a repressor of the noncanonical NF-κB pathway, acts as a potent negative regulator of LTR-dependent HIV-1 transcription [12]. Whether additional factors are involved in HIV transcription regulation through upstream NF-κB signaling remains to be investigated.

C-terminus of Hsc70-interacting protein (CHIP; also called STUB1), a cellular E3 ubiquitin ligase protein, is associated with the molecular chaperones Hsp90 and Hsp70 and functions in the context of Hsp70 and Hsc70 cochaperone complexes [13]. CHIP contains three tetratricopeptide repeat domains that are involved in protein‒protein interactions and a U-box domain. Multiple studies have demonstrated that the U-box domain of CHIP displays E3 ubiquitination ligase activity and targets a broad range of substrates for proteasomal degradation in a chaperone-dependent manner [14–20], thus making CHIP an important modulator in the processes of protein degradation, cell proliferation, and tumor progression [13,21]. Recently, CHIP was reported to promote HIV-1 Tat protein degradation via ubiquitination and subsequent proteasomal degradation, thus inhibiting Tat-mediated LTR promoter transactivation and virus production [22].

In this study, we demonstrated that CHIP modulates HIV infection by limiting viral transcription through a novel mechanism, i.e., the negative regulation of the TRAF6-NF-κB axis/signaling. Mechanistically, CHIP targets TRAF6 but not TRAF2 or p65 for degradation via the ubiquitin‒proteasome pathway, leading to the inhibition of TRAF6-mediated NF-κB signaling, subsequently resulting in the suppression of NF-κB-dependent HIV-1 LTR-driven transcription. Notably, the TPR domain together with the U-box domain of CHIP play critical roles in facilitating the proteasome-mediated degradation of TRAF6. Overall, this study reveals a new role for CHIP in repressing TRAF6-NF-κB-HIV-LTR axis activation, which leads to the suppression of HIV-1 postintegration transcription and replication and the maintenance of HIV-1 latency, with both the N-terminal TPR domain and the C-terminal U-box domain contributing to CHIP E3 ubiquitin ligase activity.

## Results

### CHIP silencing increases single-cycle HIV replication at the postintegrational transcription level

**Fig 1:**
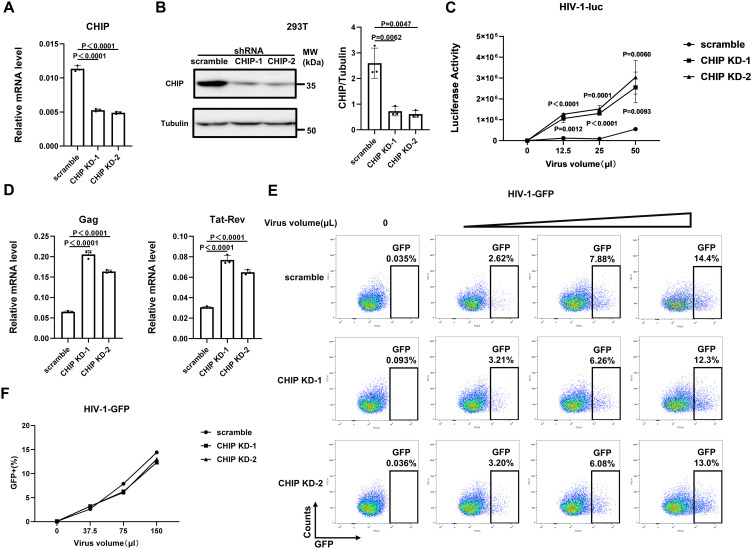
Knockdown of CHIP enhances single-cycle HIV replication at the postintegrational transcription level. **(A, B)** 293T cells were stably transduced with shRNA targeting CHIP, with cells transduced with scrambled shRNA as a control. The knockdown effect of endogenous CHIP was assessed by qPCR (**A**) and Western blot (**B**) analyses. **(C)** CHIP-KD or control 293T cells were subjected to infection with various doses (0, 12.5, 25, and 50 μl) of single-cycle VSV-G-pseudotyped NL4-3 HIV-1-luc. **(D)** CHIP-KD or control 293T cells were infected with VSV-G-pseudotyped NL4-3 HIV-1-luc for 36 h before the Gag and Tat-Rev mRNA expression were examined via qPCR. **(E, F)** CHIP-KD or control 293T cells were subjected to infection with various doses of single-cycle VSV-G-pseudotyped HIV-1-GFP virus. The percentages of GFP-positive cells were determined by flow cytometry (**E**) and are shown in a line chart **(F)**.

To gain functional insight into the potential anti-HIV-1 activity of CHIP, we used short hairpin RNA (shRNA) to generate stable 293T cell lines lacking CHIP expression (293T-CHIP-KD-1 and 293T-CHIP-KD-2), with cells stably expressing scramble shRNA (293T-scramble) as a control. Compared with those in scramble cells, the endogenous expression levels of CHIP in knockdown cell lines were strongly reduced, as evidenced by both qPCR and Western blot analyses (Fig 1A, 1B). We then infected two 293T-CHIP-KD cell lines and 293T-scramble cells with various doses of an HIV-1-luc reporter virus, i.e., a VSV-G-pseudotyped single-cycle HIV-1 pNL4–3 containing a luciferase reporter under the control of the HIV-1 LTR. Compared with scramble 293T cells, CHIP-KD 293T cells presented a 4 ~ 6-fold increase in luciferase activity (Fig 1C). Elevated mRNA levels of HIV-1 p55Gag and Tat-Rev were also observed in CHIP-KD 293T cells infected with the HIV-1-luc reporter virus (Fig 1D). These results indicate that CHIP plays an antiviral role in HIV-1 infection.

We then investigated whether CHIP restricts HIV infection in the early phase before/at integration by using HIV-1-GFP, i.e., a VSV-G-pseudotyped single-cycle HIV-1 retroviral vector with a GFP reporter under the control of a cytomegalovirus (CMV) promoter. This HIV-1-GFP pseudovirus was used to indicate the early steps from viral entry to host genome integration during HIV-1 infection. We did not observe any significant difference in the number of GFP-positive cells between the CHIP-KD 293T cells and the scramble 293T cells (Fig 1E, 1F), suggesting that CHIP has no effect on HIV infection before or at integration into the host genome. Taken together, these results indicate that CHIP negatively affects HIV replication at the postintegrational transcription level.

### CHIP limits HIV-1 gene expression and promotes HIV-1 latency in various cellular models

**Fig 2:**
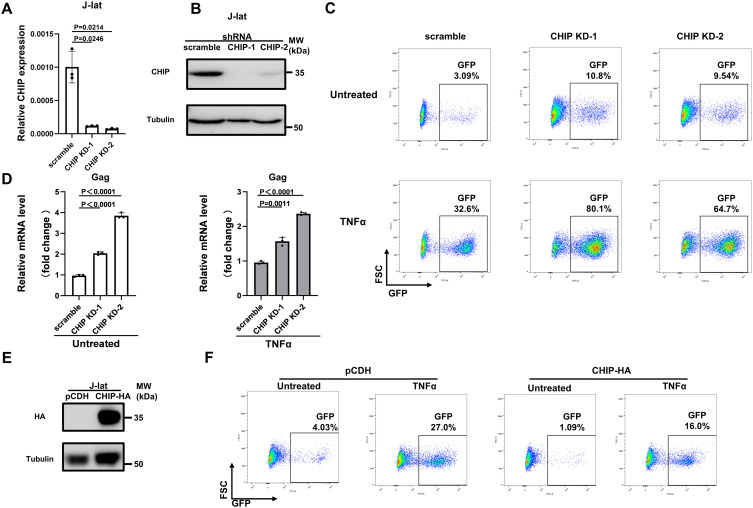
CHIP promotes HIV-1 latency in J-Lat cells. **(A, B)** J-Lat cells were stably transduced with shRNA targeting CHIP, with cells transduced with scrambled shRNA as control. The knockdown effect of endogenous CHIP was assessed by qPCR (**A**) and Western blot (**B**) analyses. **(C)** CHIP-KD or scramble J-Lat cells were treated with or without TNFα (20 ng/ml) for 24 **h.** The reactivation of HIV-1 transcription was measured by detecting GFP expression via flow cytometry. **(D)** CHIP-KD or scramble J-Lat cells were stimulated with or without TNFα (20 ng/ml) for 24 h before the Gag mRNA expression was examined via qPCR. **(E)** The protein level of ectopic expression of CHIP in J-Lat cells stably transduced with pCDH-CHIP-HA was detected by an anti-HA antibody. **(F)** J-Lat cells stably transduced with CHIP were stimulated with or without TNFα for 24 **h.** GFP expression in each group was examined to compare the HIV-1 latency reversal.

We next examined whether CHIP suppresses HIV-1 gene expression in CD4 + T cells. Chronically HIV-1-infected Jurkat CD4 + T cells (J-Lat A10.6 cells; hereafter, J-Lat cells), which harbor a full-length HIV provirus with a GFP gene in place of the *nef* gene and serve as a common cellular model for HIV-1 latency [23], were utilized. CHIP-knockdown J-Lat cells (J-Lat-CHIP-KD-1 and J-Lat-CHIP-KD-2) were generated by transducing lentiviral particles encoding shRNA targeting CHIP, with cells transduced with scramble shRNA lentivirus as a negative control. The efficiency of CHIP knockdown was confirmed by qPCR and Western blot analyses (Fig 2A, 2B). Compared with control cells, CHIP-KD J-Lat cells presented a 2 ~ 3-fold increase in LTR-driven GFP reporter expression in the absence or presence of TNFα (Fig 2C), a latency reversing agent (LRA) that is involved in NF-κB pathway-dependent HIV-1 transcription stimulation [5]. Consistent with these results, the knockdown of endogenous CHIP increased the transcription of Gag in both the absence and presence of TNFα (Fig 2D). We then engineered a J-Lat cell line with a pCDH-vector-based CHIP with an HA tag (Fig 2E). In agreement with the data obtained for CHIP-KD J-Lat cells, compared with cells transduced with empty lentivectors, the J-Lat cell line overexpressing CHIP presented reduced basal LTR-driven GFP transcription and impaired latency reactivation in the presence of TNFα (Fig 2F), suggesting that the ectopic expression of CHIP downregulates HIV-1 LTR-driven transcription.

**Fig 3:**
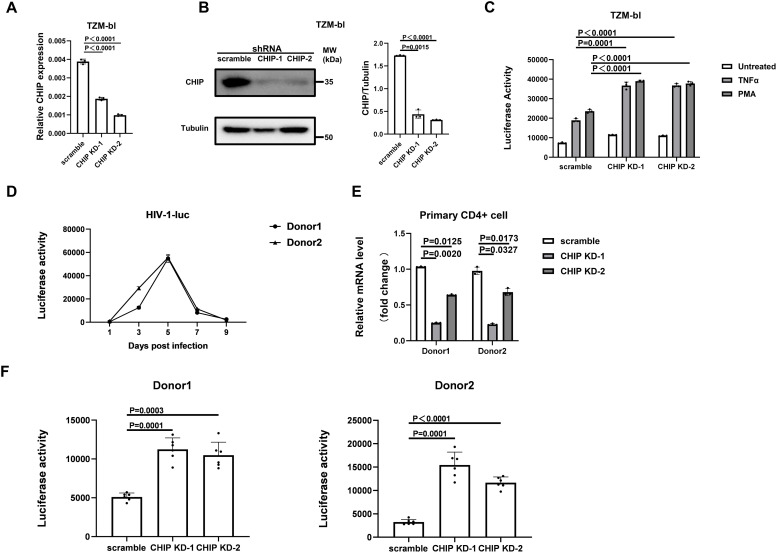
Knockdown of CHIP in TZM-bl and primary CD4  + T cells reactivates HIV-1 transcription. (**A, B**) qPCR (**A**) and Western blot (**B**) confirmed the efficient knockdown of CHIP in TZM-bl cells. **(C)** CHIP-KD or scramble-KD TZM-bl cells were seeded in 96-well plates and then treated with TNFα (20 ng/ml) or PMA (100 nM) for 24 **h.** The luciferase activity of cell lysates was then detected to indicate HIV-1transcription. Data represent at least 3 independent experiments and are presented as mean ± SD. The statistical significance analyses were performed using a two-sided unpaired t test. **(D)** Primary CD4 + T cells derived from 2 healthy donors were activated by anti-CD3/CD28 antibody-coated beads for 3 days and were then infected with a single-cycle HIV-1 pNL4-3-luc to produce a primary T cell model of latency. Cells were collected on day 1 (post-infection day of pNL4-3 luc), day 3, day 5, day 7, and day 9 to follow the development of the latency model. **(E)** At day 9 dpi, latent primary CD4 + T cells were transduced with shRNA-scramble or shRNA-CHIP lentivirus for 3 days and then selected by puromycin for another 3 days. mRNA expression level of CHIP in HIV-1 latently infected primary CD4 + T cells transduced with shRNA-scramble or shRNA-CHIP lentivirus. **(F)** Anti-CD3/CD28 antibody-coated beads were added to cells for a 3-day stimulation to reactivate HIV-1 transcription before the luciferase activity was measured.

The effect of CHIP knockdown on the reactivation of a HeLa-derived TZM-bl cell line, which contains HIV-1 integrated with an LTR-driven luciferase (luc)-reporter, was then assessed. TZM-bl-CHIP-KD cells were constructed with both shRNAs (Fig 3A, 3B). Luciferase activity was detected to assess HIV-1 transcription in TZM-bl cells treated with TNFα and PMA, another LRA that induces NF-κB-dependent HIV-1 transcription. The results revealed that CHIP knockdown increased both basal and reactivated luciferase activity in TZM-bl cells after treatment with TNFα and PMA (Fig 3C).

Furthermore, a primary CD4 + T HIV-1 latency model was established by infecting cells with a luciferase reporter-pseudotyped HIV-1 vector. The latent status of HIV-1 was successfully established (Fig 3D), followed by transduction with shRNA-CHIP or shRNA-scramble lentivectors at 7 dpi and then puromycin treatment at 10 dpi. Efficient CHIP knockdown was observed in both donors (Fig 3E). Latent HIV-1 was reactivated by anti-CD3/CD28 antibody Dynabeads at 13 dpi. Three days postreactivation, the luciferase activity in primary CD4 + T cells expressing reduced CHIP was significantly greater than that in the scramble control cells (Fig 3F).

Collectively, these data indicate that endogenous CHIP has the ability to prevent the reactivation of HIV-1 gene expression.

### CHIP suppresses HIV-1 LTR-driven gene expression via the NF-κB binding site

**Fig 4:**
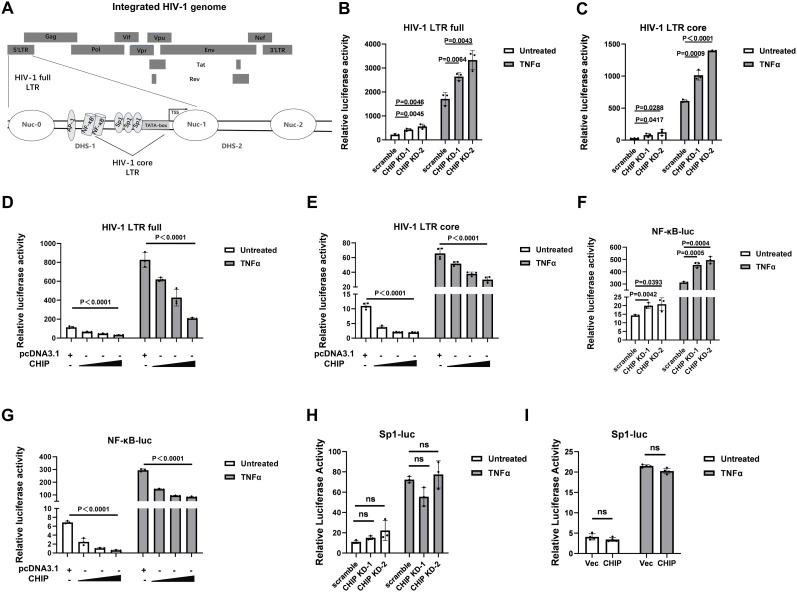
CHIP suppresses reactivation of HIV-1 LTR-driven and NF- κB-driven gene expression. **(A)** Schematic diagram of the full-length and the core elements of HIV-1 LTR that were used for constructing the luciferase reporters. **(B, C)** CHIP-KD or control 293T cells were seeded and transfected with HIV-LTR-full-luc (**B**) or HIV-LTR-core-luc **(C)**, along with pRL-TK. Twenty-four hours post-transfection, cells were treated with or without TNFα (20 ng/ml). The luciferase activity was measured at 16 h post-treatment. **(D, E)** Increasing amounts of CHIP or pCDNA3.1, HIV-LTR-full-luc (**D**) or HIV-LTR-core-luc (**E**) and pRL-TK was cotransfected in 293T cells. Twenty-four hours post-transfection, cells were treated with or without TNFα (20 ng/ml) for 16 h before the luciferase activity was examined. **(F)** NF-κB-luc reporter and pRL-TK was transfected into CHIP-KD or control scramble-KD 293T cells. Cells were treated with TNFα (20 ng/ml) for 16 **h.** The luciferase activity was determined at 24 h poststimulation using the dual luciferase reporting assay. **(G)** Increasing amounts of CHIP or pcDNA3.1, NF-κB-luc (100 ng) and pRL-TK was cotransfected in 293T cells. Twenty-four hours post-transfection, cells were treated with or without TNFα (20 ng/ml) for 16 h before the luciferase activity was examined. **(H)** CHIP-KD or control 293T cells were seeded and transfected with pGL-Sp1-luc along with pRL-TK. Twenty-four hours post-transfection, cells were treated with or without TNFα (20 ng/ml). The luciferase activity was measured at 16 h posttreatment. (**I**) pCDH-CHIP-HA or empty vector was cotransfected with pGL-Sp1-luc and pRL-TK in 293T cells. Twenty-four hours post-transfection, cells were treated with or without TNFα (20 ng/ml) for 16 h before the luciferase activity was examined.

Major determinants located in the HIV-1 5’ LTR regulate viral transcription and productive infection [3] (Fig 4A). We next investigated, by performing HIV-1 LTR-driven firefly luciferase reporter assays, whether CHIP affects the HIV-1 LTR promoter activity directly. A full-length HIV-1 LTR or a core HIV-1 LTR reporter (schematic representation in Fig 4A) was transfected into stable CHIP-KD-1 and CHIP-KD-2 293T cell lines. Compared with the scramble-shRNA-transduced 293T cells, the CHIP-KD 293T cells displayed increased luciferase activity for both LTR-full- and LTR-core-driven transcription in the absence or presence of TNFα (Fig 4B, 4C). Furthermore, CHIP overexpression suppressed basal and TNFα-induced HIV-LTR-full activity and HIV-LTR-core luciferase reporter activity in a dose-dependent manner (Fig 4D, 4E). Taken together, these data suggest that CHIP negatively regulates HIV-1 LTR-driven gene expression.

NF-κB and Sp1 binding sites play important roles in regulating the transcriptional activity of HIV-1 core LTR-driven transcription [Fig 4A, also described in [24]]. Therefore, we sought to determine whether CHIP regulates NF-κB- or Sp1-driven promoter activity. The results showed that CHIP had a direct effect on NF-κB-driven transcription, as the luciferase reporter activity of NF-κB was significantly greater in the TNFα-treated CHIP-KD 293T cells than in the control cells (Fig 4F) and the expression of NF-κB-responsive inflammatory gene (i.e., IL6 and CXCL10) was higher in the CHIP-KD cells (S1 Fig). Moreover, CHIP overexpression suppressed TNFα-induced NF-κB reporter activity in a dose-dependent manner (Fig 4G). However, no significant difference in Sp1 reporter activity was observed between CHIP-overexpressing cells or CHIP-knockdown cells and control cells (Fig 4H, 4I), suggesting that CHIP regulates the transcriptional activity of the HIV-1 LTR by targeting NF-κB-mediated but not Sp1-mediated activation.

### CHIP inhibits HIV-1-LTR transactivation in a TRAF6-NF-κB-dependent manner

**Fig 5:**
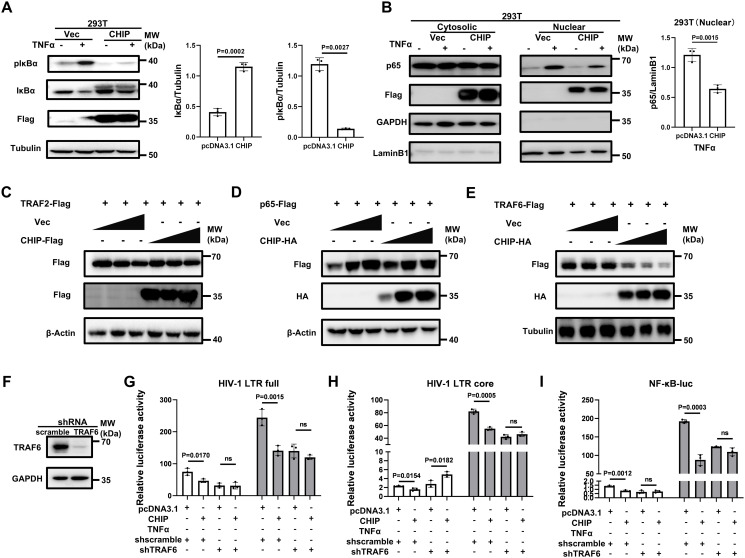
CHIP inhibits HIV-1-LTR-mediated transcription in a TRAF6-NF-κB dependent manner. **(A)** Phosphorylation and degradation of IκBα were detected in CHIP-overexpressing cells treated by TNFα (20 ng/ml) for 30 min. Immunoblotting was performed by an anti-pIκBα or an anti-IκBα antibody. **(B)** Cytoplasmic and nuclear p65 of 293T cells transfected with Flag-tagged CHIP or an empty vector treated by TNFα (20 ng/ml) for 1 h were detected. **(C–E)** Increasing amounts of plasmid encoding Flag-tagged CHIP or HA-tagged CHIP and equal amounts of plasmid expressing Flag-tagged TRAF2 **(C)**, p65 (**D**) or TRAF6 (**E**) were cotransfected into 293T cells. The expression levels of exogenous proteins were immunodetected using an anti-Flag antibody. **(F)** The knockdown effect of endogenous TRAF6 in 293T cells stably transduced with shRNA targeting TRAF6 was assessed by Western blot. (**G–I**) TRAF6-knockdown or control cells were seeded and cotransfected with CHIP or pCDNA3.1 and HIV-LTR-full-luc **(G)**, HIV-LTR-core-luc (**H**) or NF-κB-luc **(I)**, along with pRL-TK. Twenty-four hours post-transfection, cells were treated with or without TNFα (20 ng/ml). The luciferase activity was measured at 16 h posttreatment.

Upon the activation of NF-κB upstream stimuli, IKK is activated, followed by the phosphorylation and degradation of IκBα, releasing p65 and promoting its translocation into the nucleus, where it binds to the NF-κB binding site [5]. In CHIP-overexpressing cells, the TNFα-induced activation of IκBα was greatly reduced, as shown by the decrease in pIκBα and increase in the expression of IκBα (Fig 5A); NF-κB p65 nuclear translocation was significantly compromised compared with that in control cells (Fig 5B). Thus, we conclude that CHIP functions upstream of NF-κB signaling, preventing the translocation of NF-κB into the nucleus and therefore hampering HIV transcription.

Previous studies have demonstrated that CHIP displays E3 ubiquitination ligase activity and targets a broad range of substrates for proteasomal degradation, including proteins that mediate NF-κB signal transduction [14,25–27]. Hence, we sought to determine which signal-transducing factor(s) in the upstream NF-κB pathway are degraded by CHIP. The ectopic expression levels of TRAF2, TRAF6 and p65 were examined, followed by the overexpression of CHIP. The results showed that CHIP induced the dose-dependent degradation of TRAF6 but had no effect on TRAF2 or p65 protein levels under identical experimental conditions (Fig 5C–5E). Next, to determine whether CHIP-mediated HIV-1-LTR repression is dependent on TRAF6, we deleted endogenous TRAF6 to assess the regulatory effects of CHIP on the HIV-1 LTR and NF-κB-driven transcriptional activity. TRAF6 knockdown caused a significant reduction in both HIV-1 LTR and NF-κB-responsive promoter activities (Fig 5F-5I), a finding that is consistent with the ability of TRAF6 to promote NF-κB activity [28,29]. Notably, unlike scramble cells, in which CHIP overexpression potently suppressed HIV-1 LTR full-driven (~38% inhibition, p < 0.001), HIV LTR core-driven (~34% inhibition, p < 0.001) or NF-κB-driven (~48% inhibition, p < 0.001) transcription, TRAF6-deficient cells completely abrogated these inhibitory effects (Fig 5G–5I). Thus, these data suggest that TRAF6 serves as an essential mediator in CHIP-mediated HIV-1 LTR regulation.

### CHIP inhibits NF-κB-mediated HIV-1 transcription by degrading TRAF6 in a ubiquitin–proteasome-dependent manner

**Fig 6:**
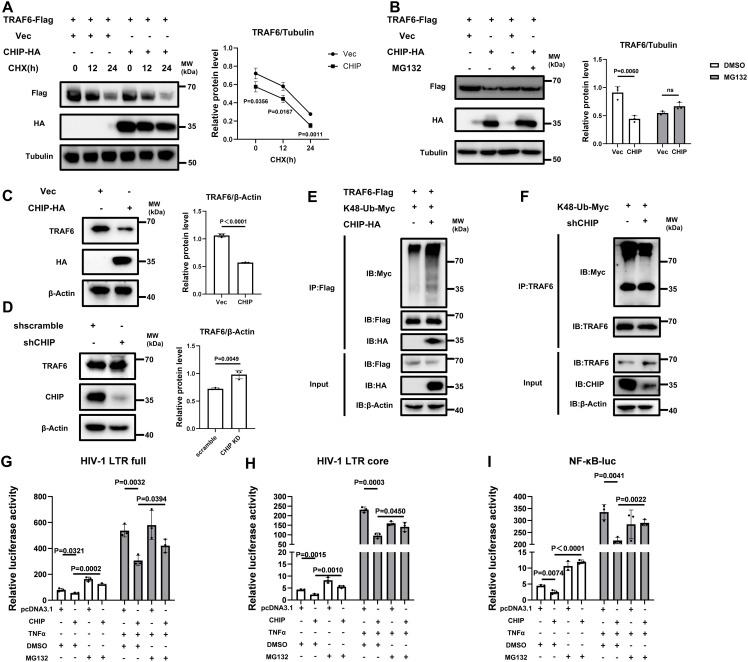
CHIP negatively regulates the NF-κB signaling pathway through promoting TRAF6 degradation via the ubiquitin–proteasome pathway. **(A)** CHX chase analyses of Flag-tagged TRAF6 in 293T cells cotransfected with HA-tagged CHIP or pCDH vector. Cells were treated with 100 μg/ml CHX for the time points indicated. The immunoblot values of TRAF6-flag expression were quantified. **(B)** 293T cells were cotransfected with Flag-tagged TRAF6 and HA-tagged CHIP or pCDH vector. Cells were treated with MG132 (20 μM) for 8 **h.** The immunoblot values of TRAF6-flag expression were quantified. **(C, D)** Endogenous TRAF6 expression in 293T cells that were transfected with HA-tagged CHIP for 48 hours (**C**) or 293T-CHIP-KD cells (**D**) was assessed by using an anti-TRAF6 antibody and the immunoblot values were quantified. **(E)** Immunoblot analysis of 293T cells cotransfected with Flag-tagged TRAF6, K48-Ub-Myc and HA-tagged CHIP or empty vector. The cell lysates were immunoprecipitated with an anti-Flag antibody and the levels of ubiquitination were measured with an anti-Myc antibody. **(F)** 293T-CHIP-KD cells were transfected with K48-Ub-Myc. Cell lysates were immunoprecipitated with an anti-TRAF6 antibody and the levels of K48 ubiquitination levels were assessed with an anti-Myc antibody. (**G–I**) 293T cells were seeded and cotransfected with CHIP or pcDNA3.1 and HIV-LTR-full-luc **(G)**, HIV-LTR-core-luc (**H**) or NF-κB-luc **(I)**, along with pRL-TK. Twenty-four hours posttransfection, cells were treated with or without TNFα (20 ng/ml). The luciferase activity was measured before treatment with MG132 (20 μM) or DMSO for 8 **h.**

We then aimed to elucidate the molecular mechanism underlying the CHIP-mediated degradation of TRAF6. Cycloheximide (CHX), which blocks protein synthesis, was used to evaluate the influence of CHIP on the protein stability of TRAF6. The CHX-chase assay results revealed that CHIP overexpression accelerated CHX-induced TRAF6 degradation (Fig 6A). Next, treatment with the proteasome inhibitor MG132 abolished CHIP-induced TRAF6 degradation (Fig 6B), suggesting that CHIP mediates TRAF6 degradation via the ubiquitin–proteasome pathway. The expression of endogenous TRAF6 decreased in response to the overexpression of CHIP but increased in response to the knockdown of endogenous CHIP (Fig 6C, 6D). Furthermore, immunoprecipitation experiments revealed an increased number of K48-linked polyubiquitin chains on TRAF6 in the presence of ectopic CHIP (Fig 6E), while K48-Ub modification of endogenous TRAF6 protein was reduced by the knockdown of endogenous CHIP (Fig 6F), demonstrating that CHIP regulates the K48-linked polyubiquitination of TRAF6. To assess the functional relevance of this mechanism, we evaluated the impact of MG132 on CHIP-mediated HIV-1 suppression and observed a significant but partial loss of the suppression of HIV-1 LTR promoter activity and NF-κB-luc activity caused by CHIP in the presence of MG132 treatment (Fig 6G–6I).

Taken together, these results indicate that the inhibition of LTR activity by CHIP is primarily due to the suppression of TRAF6-NF-κB axis activation via the promotion of TRAF6 degradation through the ubiquitin–proteasome pathway.

### The TPR domain and the U-box domain of CHIP independently degrade TRAF6

**Fig 7:**
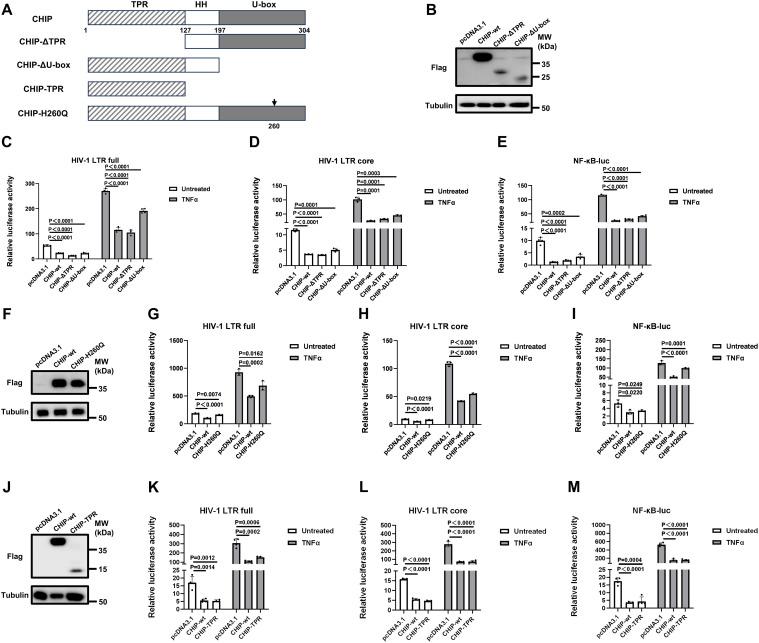
The TPR domain and the U-box domain are critical in promoting CHIP’s transcriptional regulation on HIV-1 LTR and NF-κB promoter activities. **(A)** Structural schematic diagram illustrating the wild-type, domain-truncated mutants, and functionally deficient point mutant of CHIP. (**B)** Western blot that confirms the expression of wild-type CHIP, CHIP-ΔTPR or CHIP-ΔU-box. **(C–E)** 293T cells were seeded and cotransfected with pcDNA3.1, wild-type CHIP, CHIP-ΔTPR or CHIP-ΔU-box and HIV-LTR-full-luc **(C)**, HIV-LTR-core-luc (**D**) or NF-κB-luc **(E)**, along with pRL-TK. Twenty-four hours post-transfection, cells were treated with or without TNFα (20 ng/ml). The luciferase activity was measured at 16 h posttreatment. **(F, J)** Western blot that confirms the expression of CHIP-H260Q mutant (**F**) and CHIP-TPR **(J)**. (**G–I, K–M**) 293T cells were seeded and cotransfected with pcDNA3.1, wild-type CHIP, CHIP-H260Q or CHIP-TPR and HIV-LTR-full-luc **(G, K)**, HIV-LTR-core-luc (**H, L**) or NF-κB-luc **(I, M)**, along with pRL-TK. Twenty-four hours post-transfection, cells were treated with or without TNFα (20 ng/ml). The luciferase activity was measured at 16 h posttreatment.

CHIP is composed of an N-terminal TPR domain, a C-terminal U-box domain, and a linker region connecting these two domains, among which the U-box domain is well recognized to facilitate ubiquitin transfer for ubiquitination [21]. To determine the critical domain(s) mediating the regulatory function of CHIP in HIV-1 transcription, we constructed a series of CHIP domain-truncation mutants (Fig 7A). Dual-luciferase reporter assays demonstrated that, as with wild-type CHIP, the ΔTPR mutant retained strong inhibitory effects against the HIV-1 LTR full, the HIV-1 LTR core, and NF-κB-responsive promoters (Fig 7B–7E), suggesting that the U-box domain and the middle linker region are responsible for the regulatory function of CHIP. However, for the ΔU-box mutant, inhibitory activity against the HIV-1 LTR full-luc, HIV-1 LTR core-luc and NF-κB-luc promoters was only partially lost (Fig 7C–7E). Notably, the inhibitory effects of the ΔTPR mutant and the ΔU-box mutant were comparable even when the expression was much lower than that of wild-type CHIP, indicating that the protein instability of these two mutants was not related to their HIV-1-suppression activity. Next, histidine residue 260 (H260), which is responsible for U-box domain E3 ligase activity [14], was mutated to H260Q (Fig 7F) to validate the results obtained with the ΔU-box mutant. Although the efficacy of CHIP-H260Q was lower than that of wild-type CHIP, all reporter activities were significantly suppressed (Fig 7G–7I), indicating that the U-box domain is critical but not sufficient for the CHIP-mediated regulation of NF-κB signaling and HIV-1 transcriptional activation.

As the above data suggest that other domains of CHIP may contribute to its HIV-1 LTR and NF-κB suppression, a mutant that contains only a TPR domain was subsequently constructed (Fig 7J). Fig 7K–7M shows that the TPR domain alone exhibited inhibitory potency comparable to that of wild-type CHIP, although CHIP-TPR was expressed at a lower level than wild-type CHIP, suggesting a greater specific activity. Combined with the results in Fig 7C–7E showing that CHIP devoid of the TPR domain retained strong inhibitory effects, we conclude that the TPR domain and the U-box domain play independent roles in mediating the transcriptional regulation function of CHIP.

**Fig 8:**
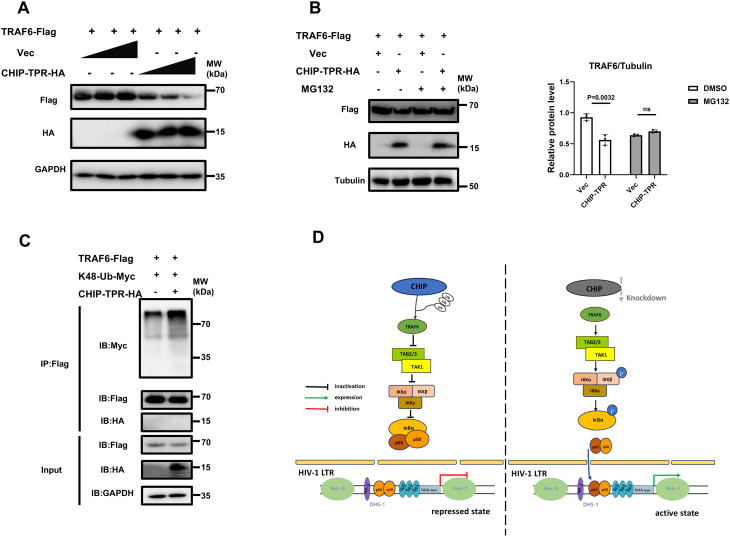
The TPR domain mediates ubiquitin-dependent degradation of TRAF6. **(A)** Increasing amounts of plasmid encoding HA-tagged CHIP-TPR and equal amounts of plasmid expressing Flag-tagged TRAF6 were cotransfected into 293T cells. The expression levels of exogenous proteins were immunodetected using an anti-Flag antibody. **(B)** 293T cells were cotransfected with Flag-tagged TRAF6 and HA-tagged CHIP-TPR or pCDH vector. Cells were treated with MG132 (20 μM) for 8 **h. (C)** Immunoblot analysis of 293T cells cotransfected with Flag-tagged TRAF6, K48-Ub-Myc and HA-tagged CHIP-TPR or empty vector. The cell lysates were immunoprecipitated with an anti-Flag antibody and the levels of ubiquitination were measured with an anti-Myc antibody. **(D)** A proposed schematic mechanism for CHIP -mediated suppression of HIV-1 transcription and latent reactivation.

While the TPR domain of CHIP has been shown to mediate interactions with chaperones [21], there are also studies reporting that it facilitates protein degradation [30]. Thus, we assessed whether the TPR domain is necessary for the degradation of TRAF6. The results showed that the TPR domain downregulated TRAF6 expression in a dose-dependent manner (Fig 8A) and that the downregulatory effect on TRAF6 was abolished by treatment with MG132 (Fig 8B). In addition, the overexpression of the TPR domain promoted the K48-linked polyubiquitination of TRAF6 (Fig 8C), indicating that the TPR domain alone contributes to TRAF6 degradation via the ubiquitin–proteasome pathway (Fig 8D).

## Discussion

The role of CHIP in regulating the NF-κB signaling pathway has been implicated in tumorigenesis, osteoclast formation, neurological disorders, and innate immunity [31–34]; however, there are no reports describing its participation in the regulation of NF-κB signaling in viral infection or viral transcription. Here, we show that CHIP potently represses HIV-1 LTR-directed transcription and reverses viral latency by negatively modulating TRAF6-NF-κB axis signaling. Notably, we demonstrated that the TPR domain and the U-box domain of CHIP function independently in mediating TRAF6 degradation via the ubiquitin–proteasome pathway.

The postintegration transcription of HIV-1 is tightly regulated by the interaction of numerous host proteins with cis-acting DNA sequences present on the LTR promoter of the provirus. The major regulatory sites in the LTR promoter are ‘core promoter’ and ‘core enhancer’ sites. The ‘core promoter’ contains a TATA box and three binding sites for Sp1, whereas the ‘core enhancer’ has two NF-κB binding sites. The establishment, maintenance, and reactivation of HIV-1 latency are correlated with the activation levels of NF-κB. Upstream of the NF-κB-mediated LTR transcription activation, multiple proteins, including TRAF family members, are responsible for NF-κB signal transduction. Among TRAFs, TRAF6 generates free K63-polyubiquitin chains that bind to TAB2/3 to activate TAK1 and bind to NEMO to activate IKKα/β in the receptor complex, ultimately resulting in NF-κB activation [6]. Our data revealed that TRAF6 knockdown caused a 36% decrease in TNF-α-induced NF-κB reporter activation and a 40% decrease in HIV-1 LTR promoter activation (Fig 5G–5I). Although reports have shown that CHIP promotes the ubiquitination and degradation of NF-κB p65 and TRAF2 [25,32], our data in this study and others [31] failed to reveal such effects. Instead, we detected a strong degradation effect of CHIP on TRAF6.

CHIP contains a U-box domain in the C-terminus, a coiled-coil region in the middle, and a TPR domain in the N-terminus. With the U-box domain attributed to the activity of E3 ubiquitin ligases, CHIP mediates the degradation of target proteins and contributes to the quality control of proteins and cell fate determination; thus, CHIP is a modulator of various physiological processes as well as diverse pathological conditions, including neurodegenerative diseases, cardiovascular diseases and tumors [31–34]. CHIP has also been reported to inhibit Tat-mediated HIV-1 LTR promoter transactivation by promoting Tat protein degradation via polyubiquitination [22]. However, we did not observe any downregulation effect on HIV-1 Tat caused by the overexpression of CHIP (S2 Fig). In agreement with previous studies demonstrating that the U-box domain has E3 ligase activity, our data showed that CHIP promoted the K48 ubiquitination of TRAF6. Although we observed the significant loss of CHIP-mediated HIV-1 suppression when MG132 treatment was used to block TRAF6 ubiquitination (Fig 6G–6I), MG132 failed to induce the total abrogation of CHIP activity, indicating that CHIP may utilize other strategies to regulate TRAF6, which needs further investigation.

Furthermore, we found that the U-box domain is not sufficient and that the TPR domain also contributes to TRAF6 degradation and CHIP-mediated HIV-1 LTR suppression (Fig 7C–7E). Although the TPR domain is well known to mediate protein‒protein interactions [21], there are also reports suggesting that it may be involved in other processes. For example, the TPR domain but not the U-box domain has been shown to be required for CHIP to promote NIK degradation [30]. The observation in this study that the TPR domain did not interact with TRAF6 but the K48-linked ubiquitination of TRAF6 still occurred (Fig 8C) may be due to the interaction of TRAF6 with other proteins. Hsp70, the main heat shock chaperone associated with CHIP, has been reported to inhibit HIV-1 gene expression and replication [35]. We investigated whether CHIP regulated the HIV-1 LTR by interacting with Hsp70 by utilizing CHIP K30A, a mutation that blocks the association between CHIP and Hsp70 [13,36]. The results revealed that CHIP K30A strongly inhibited LTR activity, similar to wild-type CHIP (S3 Fig), suggesting that CHIP regulates the HIV-1 LTR in an Hsp70-independent manner.

In summary, we identified the critical role of CHIP in repressing HIV-1 postintegration transcription and facilitating the maintenance of latent HIV-1 in a TRAF6-NF-κB-dependent manner. Theoretically, targeting CHIP may help diminish HIV-1 latency and increase the efficiency of HIV-1 latency reversal agents. Therefore, our findings may provide a new therapeutic target for combating HIV infection and latency.

## Materials and methods

### Ethics statement

The study was conducted under local ethical regulation after approval by the Medical Ethics Review Committee of Institute of Life Sciences, Kunming University of Science and Technology (Approval number: KMUST-MEC-226). Informed consent was obtained from each healthy donor before the study.

### Reagents and antibodies

Reagents used in this study are as follows: TNF-α (10602-HNAE; Sino Biological Inc.), phorbol 12-myristate 13-acetate (PMA; P8139; Sigma-Aldrich), Puromycin (ant-pr-1; Invivogen), Dynabeads Human T-Activator CD3/CD28 (CD3/28-Dynabeads) (11161D; Thermo Fisher Scientific), CD4 MicroBeads (130-045-101; Miltenyi Biotec), Cycloheximide (CHX; S7418; Selleck), Proteasome inhibitor MG132 (HY-13259; MCE).

Mouse anti-HA (201113), rabbit anti-CHIP (R25823), rabbit anti-TRAF6 (R25966) and mouse anti-β-Actin (200068-8F10) were from Zen-Bioscience. Rabbit anti-HA(H6908) and Mouse anti-Flag (F1804) were from Sigma-Aldrich. Mouse anti-GAPDH (60004–1), Mouse anti-Tubulin (66031–1), rabbit anti-Flag (20543–1), mouse anti-GFP (50430–2), mouse anti-Myc (60003–2), rabbit anti-Myc (16286–1), goat anti-mouse IgG-HRP and goat anti-rabbit IgG-HRP were from Proteintech. Rabbit anti-IκBα (4814), anti-phospho-IκBα (2859), anti-NF-κB p65 (8242) were purchased from Cell Signaling Technology. Rabbit anti-LaminB1 antibody (AF5222) was from Beyotime Biotec.

### Cell culture

Human embryonic kidney 293T cells and TZM-bl cells were maintained in Dulbecco’s modified Eagle’s medium (DMEM; Gibco) supplemented with 10% certified fetal bovine serum (FBS; Vazyme, China). HIV-1 latently infected Jurkat T cells J-Lat A10.6 (J-Lat) were grown in Roswell Park Memorial Institute-1640 (RPMI-1640; Gibco) with 10% FBS. All cell lines were tested mycoplasma-free by a mycoplasma detection kit (Beyotime) periodically.

Healthy human donors’ peripheral blood mononuclear cells (PBMCs) were isolated from the buffy coat as previously described [10]. Naive CD4 + T cells were isolated from PBMCs by MACS microbead-negative sorting and the naive CD4 + T-cell isolation kit (Miltenyi Biotec). Primary CD4 + T-cells were maintained in RPMI-1640 with 10% FBS (Gibco) containing 30 U/ml recombinant human interleukin-2 (IL-2; Sino Biological Inc.).

### Plasmid construction and virus production

The pcDNA3.1 was obtained from NIH AIDS reagent program. CHIP sequence was cloned from human 293T cDNA by primers: HindⅢ-CHIP-For: 5’-CCCAAGCTTGCCACCATGAAGGGCAAGGAGGAGAAGGAGG-3’ and CHIP-NotⅠ-Rev: 5’-TTGCGGCCGCTCAGTAGTCCTCCACCCAGCCATTC-3’ and then ligated to pcDNA3.1-3 × flag. CHIP sequence was then subcloned into the pCDH-EF1-MCS-T2A-puro lentivector to make pCDH-CHIP-HA.

Domain-deletion and point mutant constructs of CHIP were generated by overlapping PCR using primers as follow: CHIP-deltaTPR-For: 5’-CCCAAGCTTGCCACCATGCGGCTGAACTTCGGGGACG-3’, CHIP-deltaTPR-Rev: 5’-TTGCGGCCGCTCAGTAGTCCTCCACCCAGCCATTC-3’, CHIP-deltaU-box-For: 5’-CCCAAGCTTGCCACCATGAAGGGCAAGGAGGAGAAGGAGG-3’, CHIP-deltaU-box-Rev: 5’-ATTTGCGGCCGCCTAGGCCTCAATGCAGGC-3’, CHIP-H260Q-For: 5’-GACATCGAGGAGCAACTGCAGCGTGTG-3’, CHIP-H260Q-Rev: 5’-CACACGCTGCAGTTGCTCCTCGATGTC-3’, CHIP-K30A-For: 5’-GCGCAGGAGCTCGCAGAGCAGGGCAATCGTCTGTT-3’, CHIP-K30A-Rev: 5’-AACAGACGATTGCCCTGCTCTGCGAGCTCCTGCGC-3’, CHIP-TPR-NotⅠ-Rev: 5’-ATTTGCGGCCGCCTACTGCTCCTTGGCCAGGCTGT-3’, CHIP-TPR-HA-Rev: 5’-AGTCTGGGACGTCGTATGGGTAGCCGCCCTGCTCCTTGGCC-3’, HA-NotI-Rev： 5’-TTTTCCTTTTGCGGCCGCTCAAGCGTAGTCTGGGAC-3’.

Short hairpin RNA (shRNA) cloned into pLKO.1 are as follow: scramble: 5’-CCTAAGGTTAAGTCGCCCTCG-3’. CHIP-1: 5’-GCTGGAGATGGAGAGCTATGA-3’. CHIP-2: 5’-CCAGCTGGAGATGGAGAGTTA-3’. TRAF6: 5’-TATCTCAGAGGTCCGGAATTT-3’.

HIV-1 Tat was amplified from pNL4–3-luc + env-rev- and then cloned into pEGFP-C3 expression vector. pGL4.32-NF-κB-luc (containing a 3* NF-κB responsive sites) and pRL-TK (*Renilla* luciferase) reporter plasmids were purchased from Promega. pGL4.32-Sp1-luc was constructed by inserting a synthesized 3* Sp1 responsive sequence into pGL4.32 at the NheI and BglII restriction sites. pGL4.32-LTR-core-luc and pGL4.32-LTR-full-luc were cloned in our previous study [10].

HIV-1-GFP was produced by co-transfecting pMD2.G, psPAX2 and TRIP-GFP at a 1:3:4 ratio. VSVG-pNL4–3-luc was produced by co-transfecting pMD2.G and pNL4–3-luc^+^env^-^rev^-^ at a 1:4 ratio. The transfection was performed using PEI (Polysciences).

### Generation of stably transduced cell lines

HEK293T, J-Lat, TZM-bl cells were transduced with shRNA virus and cultured for 48 h followed by puromycin selection. The supernatant of infected cells was replaced with fresh DMEM or RPMI-1640 and infected cells were cultured for another 2 days. J-Lat-CHIP-HA cells were generated by transducing pCDH-CHIP-HA lentivirus 1 μg/ml puromycin were used for selection. The knockdown efficiency was confirmed by Western blot and Sanger sequencing and the expression of CHIP was detected by anti-HA antibodies.

### Flow cytometry

For HIV-1 replication cycle assays, 293T cells were seeded in 12-well plates at a density of 2 × 10^5^ cells/ml and infected with HIV-1-GFP lentiviral. Forty-eight hours post-infection, 293T cells were washed with phosphate-buffered saline (PBS) and fixed by 4% paraformaldehyde (PFA) for flow cytometry. The percentage of GFP-positive cells was examined on a BD FACSverse cytometer driven by FACSuite 1.0.3 (BD). Analysis of the acquired data was performed by FlowJo 10 (TreeStar).

### Infection, transfection and reporter assays

293T cells (2 × 10^4^) were seeded in 96-well plates one day before infected with a single-cycle HIV-1 pNL4–3 containing a luc reporter pseudotyped with VSV-G. Forty-eight hours post-transfection, 293T cells were collected and luciferase reporter assays were performed according to the protocol of luciferase reporter assay kit (Beyotime).

293T cells (2 × 10^4^) were seeded in 96-well plates one day before cotransfected with a viral LTR-driven luciferase construct, pRL-TK, and CHIP-WT or CHIP-mutant-expressing plasmid using Lipofectamine 2000 (Invitrogen, 11668–019). Twenty-four hours post-transfection, 293T cells were left untreated or treated with TNFα (20 ng/ml) for 24 h before luciferase reporter assays were performed according to the protocol of dual luciferase reporter assay kit (Promega).

### Cycloheximide chase assays

293T cells (2 × 10^5^) were seeded in 12-well plates one day before treatment with 100 μg/ml CHX (Selleck) and incubated at 37 ◦C for the indicated time. Cells were lysed and equal amounts of cell extracts were subjected to immunoblotting.

### Real-time quantitative PCR

Total cellular RNA was extracted using a SteadyPure Quick RNA Extraction Kit (Accurate Biology) and reverse transcribed into cDNA using the Evo M-MLV RT Mix Kit with gDNA Clean for qPCR Ver.2 (Accurate Biology). Real-time PCR was performed using the SYBR Green Premix Pro Taq HS qPCR Kit (Rox Plus) (Accurate Biology) on the Bio-Rad CFX96 real-time PCR system. The primers used were: CHIP, forward, 5’-CTCAAGGAGCAGGGAAACCG-3’, and reverse, 5’- GGAAGAAGTGCGCCTTCACA-3’; Gag, forward, 5’-GTGTGGAAAATCTCTAGCAGTGG-3’, and reverse, 5’-CGCTCTCGCACCCATCTC-3’; Tat-Rev, forward, 5’-ATGGCAGGAAGAAGCGGAG-3’, and reverse, 5’-ATTCCTTCGGGCCTGTCG-3’; and GAPDH, forward, 5’-ATCCCATCACCATCTTCCAGG-3’, and reverse, 5’-CCTTCTCCATGGTGGTGAAGAC-3’; CXCL10 forward, 5’-TCCACGTGTTGAGATCATTGC-3’ and reverse, 5’-TCTTGATGGCCTTCGATTCTG-3’; IL6 forward, 5’- ACTCACCTCTTCAGAACGAATTG-3’ and reverse, 5’- CCATCTTTGGAAGGTTCAGGTTG-3’. Amplifications were run as follows: initial activation was at 95 °C for 2 min, and the subsequent 40 cycles in two phases consisted of 95 °C for 15 s and 60 °C for 30 s. The expression level was normalized with GAPDH.

### Western blot analyses and coimmunoprecipitation

Whole-cell lysates were obtained using RIPA lysis buffer (20 mM Tris-HCl pH7.5, 150 mM NaCl, 1% Triton X-100) supplemented with 1 mM phenylmethylsulfonyl fluoride (Beyotime) and protease inhibitor cocktail (Thermo Fisher Scientific). The lysates were resolved through SDS-polyacrylamide gel electrophoresis (SDS-PAGE) and subsequently transferred onto PVDF membranes (Millipore). Immunoblotting was performed using primary antibody followed by horseradish peroxidase-conjugated secondary antibody. Protein signals were visualized using chemiluminescent detection reagents (Millipore).

For nuclear and cytoplasmic fractionation, 293T cells transfected with the indicated plasmids for 36 hours were processed using Thermo Scientific NE-PER Nuclear and Cytoplasmic Extraction Reagents (78833; Thermo Fisher Scientific, USA) according to the manufacturer’s protocol.

For coimmunoprecipitation analysis, cells cultured in 6-cm dishes were lysed with 500 μl ice-cold RIPA buffer containing 1 mM PMSF and protease inhibitors. After centrifugation at 12,000 × rpm for 10 min, 450 μl supernatant was subjected to immunoprecipitation while the remaining 50 μl lysate was mixed with 5 × SDS-PAGE loading buffer, boiled for 5 min, and stored at -80°C. Protein G-agarose beads (25 μl; MCE) were pre-conjugated with 2 μg rabbit anti-Flag antibody or control IgG (CST) through 2 h incubation at 4°C. The antibody-bead complexes were washed four times with PBST (450 μl per wash) before adding 450 μl cell extract for 2 h incubation at 4°C. Following four additional PBST washes, bound proteins were eluted in 60 μl 1 × SDS-PAGE loading buffer.

### HIV-1 latently infected primary CD4 + T cell model

HIV-1 latency was established as previously described with modifications. PBMCs were isolated from leukocyte concentrates of two healthy donors via Ficoll-Paque density gradient centrifugation (Cytiva). Naïve CD4 + T cells were purified using a human naïve CD4 + T Cell Isolation Kit (Miltenyi Biotec). Following 72-hour activation with CD3/CD28 Dynabeads (Thermo Fisher Scientific), cells were maintained in IL-2-supplemented medium (30 IU/ml) for 4 days. Activated CD4 + T cells were infected with NL4–3/Luc pseudovirus, washed with pre-warmed RPMI-1640 containing IL-2 every forty-eight hours.

At 7 days post-infection (dpi), cells were transduced with either shRNA-scramble or shRNA-CHIP lentiviral vectors. Puromycin selection (500 ng/ml) was initiated at 10 dpi. Knockdown efficiency of CHIP was verified by real time PCR at 13 dpi prior to latency reversal using CD3/CD28 Dynabeads. Three days post-reactivation, cells were lysed in 100 μl passive lysis buffer for luciferase quantification. Luciferase activity was measured by mixing equal volumes of lysate and substrate (Luciferase Assay System, Promega) using a GloMax luminometer.

All human blood samples were obtained from consenting donors under protocols approved by the Kunming University of Science and Technology Institutional Review Board. Written informed consent was obtained in accordance with National Health and Medical Research Council guidelines prior to sample collection.

### Statistical analyses

Statistical analyses were done using GraphPad Prism9.0.0 software. Data are presented as means ± SD. Statistical significance between groups was assessed through two-tailed unpaired Student’s t-test (*P* values). One-way ANOVA was conducted to compare the means across different dose groups. Following the identification of a significant overall effect, a test for linear trends was applied as a post-hoc analysis to specifically evaluate the dose-dependent relationship.

## Supporting information

S1 Fig CHIP mediates NF-κB-responsive gene expression. (**A–C**) mRNA expression of CHIP (**A**), IL6 (**B**) and CXCL10 (**C**) in J-Lat-CHIP-KD cells treated with or without TNFα (20 ng/ml) was assessed via qPCR.
(TIF)

S2 Fig CHIP has no effect on HIV-1 Tat expression. Increasing amounts of plasmid encoding Flag-tagged CHIP and equal amounts of plasmids expressing GFP-tagged Tat were cotransfected into 293T cells. The expression level of exogenous Tat was assessed by immunoblotting using an anti-GFP antibody.
(TIF)

S3 Fig CHIP K30A inhibits HIV-1-LTR- and NF-κB-mediated transcription. (**A**) The expression of CHIP K30A and wild-type CHIP were detected by an anti-Flag antibody. (**B–D**) 293T cells were seeded and cotransfected with wild-type CHIP, CHIP K30A or empty vector and HIV-LTR-full-luc (**B**), HIV-LTR-core-luc (**C**) or NF-κB-luc (**D**), along with pRL-TK. Twenty-four hours posttransfection, cells were treated with or without TNFα (20 ng/ml). The luciferase activity was measured at 16 h posttreatment.
(TIF)
