# Supplementary figures and images for: CHIP/STUB1 suppresses the transcription and latent reactivation of HIV-1 via the TRAF6-NF-κB-HIV-LTR axis
Source: PLoS Pathog. 2025 Nov 7;21(11):e1013683. doi: 10.1371/journal.ppat.1013683 (PMC12617934; doi:10.1371/journal.ppat.1013683)

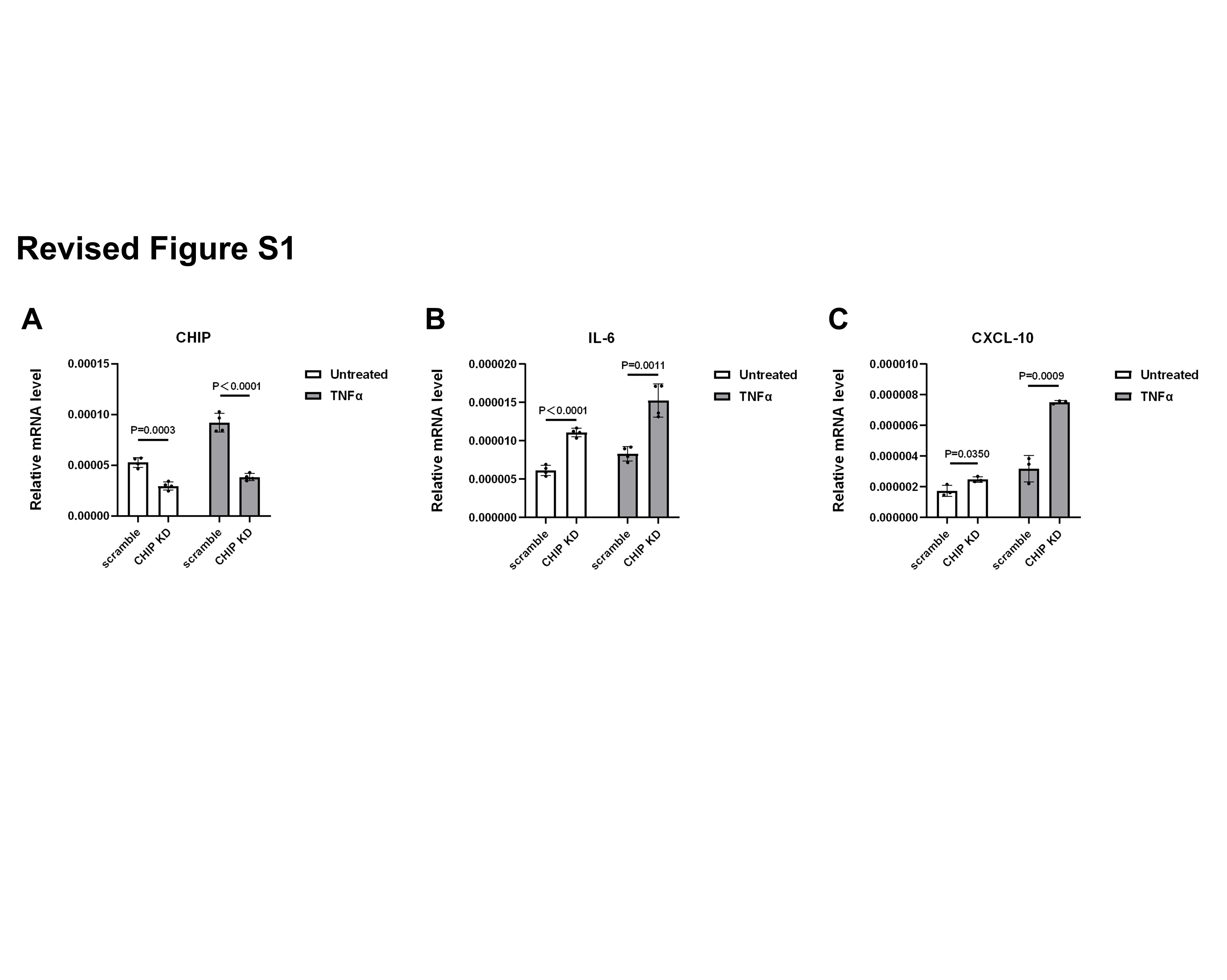

Supplement: S1 Fig — (A–C) mRNA expression of CHIP (A), IL6 (B) and CXCL10 (C) in J-Lat-CHIP-KD cells treated with or without TNFα (20 ng/ml) was assessed via qPCR. (TIF) [file ppat.1013683.s001.tif]

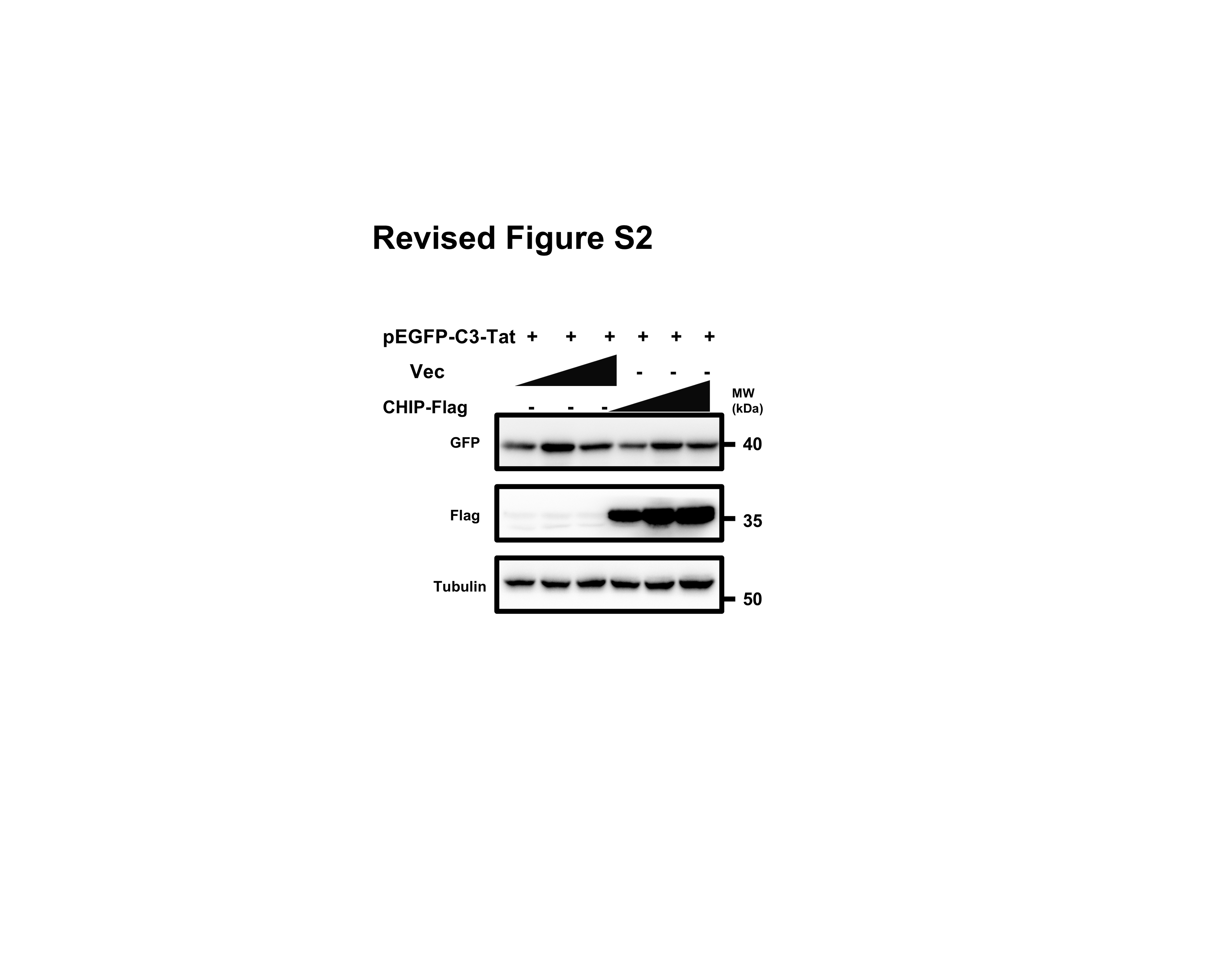

Supplement: S2 Fig — Increasing amounts of plasmid encoding Flag-tagged CHIP and equal amounts of plasmids expressing GFP-tagged Tat were cotransfected into 293T cells. The expression level of exogenous Tat was assessed by immunoblotting using an anti-GFP antibody. (TIF) [file ppat.1013683.s002.tif]

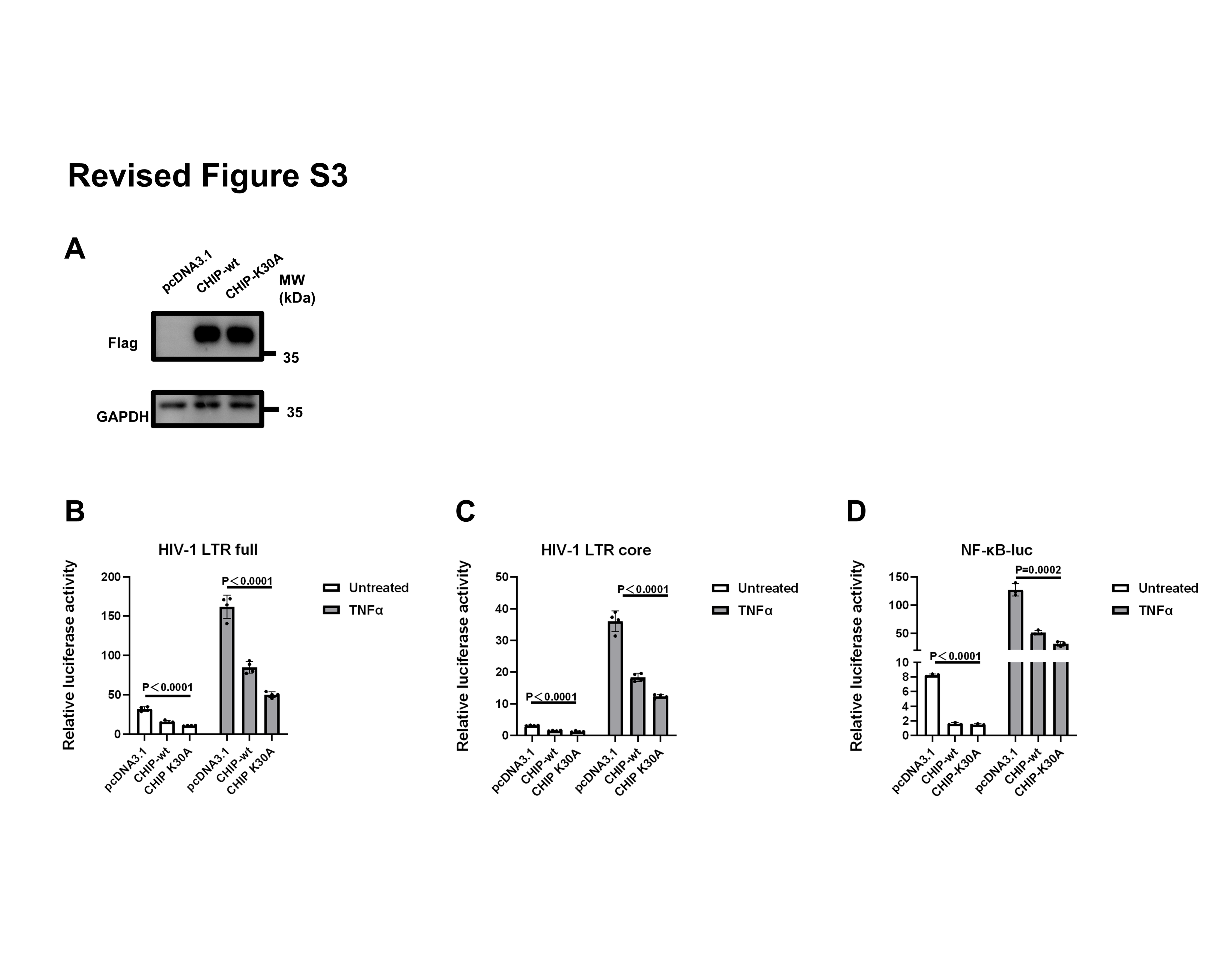

Supplement: S3 Fig — (A) The expression of CHIP K30A and wild-type CHIP were detected by an anti-Flag antibody. (B–D) 293T cells were seeded and cotransfected with wild-type CHIP, CHIP K30A or empty vector and HIV-LTR-full-luc (B), HIV-LTR-core-luc (C) or NF-κB-luc (D), along with pRL-TK. Twenty-four hours posttransfection, cells were treated with or without TNFα (20 ng/ml). The luciferase activity was measured at 16 h posttreatment. (TIF) [file ppat.1013683.s003.tif]
